# Lucy and Jupiter – understanding the planetary origins

**DOI:** 10.1038/s41467-021-27495-y

**Published:** 2021-12-15

## Abstract

The Trojan asteroids in Jupiter’s orbit have been preserved unaltered since the early ages of our Solar System. In October 2021, NASA launched its mission, *Lucy*, to visit and study these asteroids in order to learn more about the original building blocks that formed our planets.

In October 2021, to much excitement, NASA launched its *Lucy* mission to explore the Trojan asteroids, named after Trojan warriors from Homer’s *Iliad*. These asteroids are organized in two distinct clusters in Jupiter’s orbit. The *Lucy* mission is inspired by some of mankind’s most fundamental questions: how were the planets formed during the early ages of the Solar System? What made Earth habitable? And what is our place in the Solar System? Some aspects of these questions have been answered in more or less detail, some are currently under investigation and some seem too elusive to be answered in the very near future. So where does the *Lucy* mission fit into this? The Trojan asteroids are thought to be leftover parts of original building blocks that later formed the planets of our Solar System. They have been preserved unaltered since the very beginning of when our Solar System started to form^1^, and hence offer a unique possibility to gather important information about its history and evolution.

Our generation was born too late to explore Earth, and too early to explore space—yet it feels we might be just in the perfect sweet spot to explore our Solar System origins.

*Lucy* is set to visit both asteroid clusters and seven of their objects in total. It was long thought that the Trojans formed near Jupiter’s orbital distance and hence represented the original, local nebula composition of the early Solar System^1^. This would in turn mean that all Trojans show similar physical characteristics, but instead it is now known that they are physically diverse. Long range, Earth based analysis has shown that their physical composition reflects properties of objects scattered across half the outer Solar System, such as the Centaurs and the small transneptunian objects^2,3^. Ultimately, such evidence suggests that the Trojan asteroids were generated over a broad range of heliocentric distances rather than only Jupiter’s orbit, and that they will reflect a large variety of physical and compositional conditions of the original Solar System nebula^4^. In order to investigate the Trojans and collect necessary data, *Lucy* is equipped with instrumentation to explore the targets’ interior structures, bulk properties, surface composition and thermal properties^5^. Due to the Trojans’ history, the obtained data will provide us with a broad window to glimpse into the chemical and physical conditions of the nebula that formed our Solar System.

This is not the first time the name *Lucy* comes up in questions of origin. In 1974, AL 288-1, or more commonly known as *Lucy*, was discovered in Ethiopia and is the skeleton of the 3.2-million-year-old hominin species *Australopithecus afarensis*. It is the early evidence of bipedal and upright walking-gait, similar to that of humans^6^. Although the skeleton is named after the Beatles’ song *Lucy in the Sky with Diamonds*, which was playing in the camp on the evening of the skeleton’s discovery^6^, one might wonder about a possible deeper meaning. *Lucius*, the Latin origin of the name *Lucy*, means ‘light bringer’, or ‘born at the dawn of light’, which is quite appropriate for the first upright walking skeleton. It makes only sense that NASA adopted the program name *Lucy* from paleoanthropology. Just like the *Lucy* skeleton marks one of the earliest direct predecessors of humans, the Trojan asteroids may provide the earliest evidence of how our Solar System and its planets, including Earth, formed.

**Figure:**
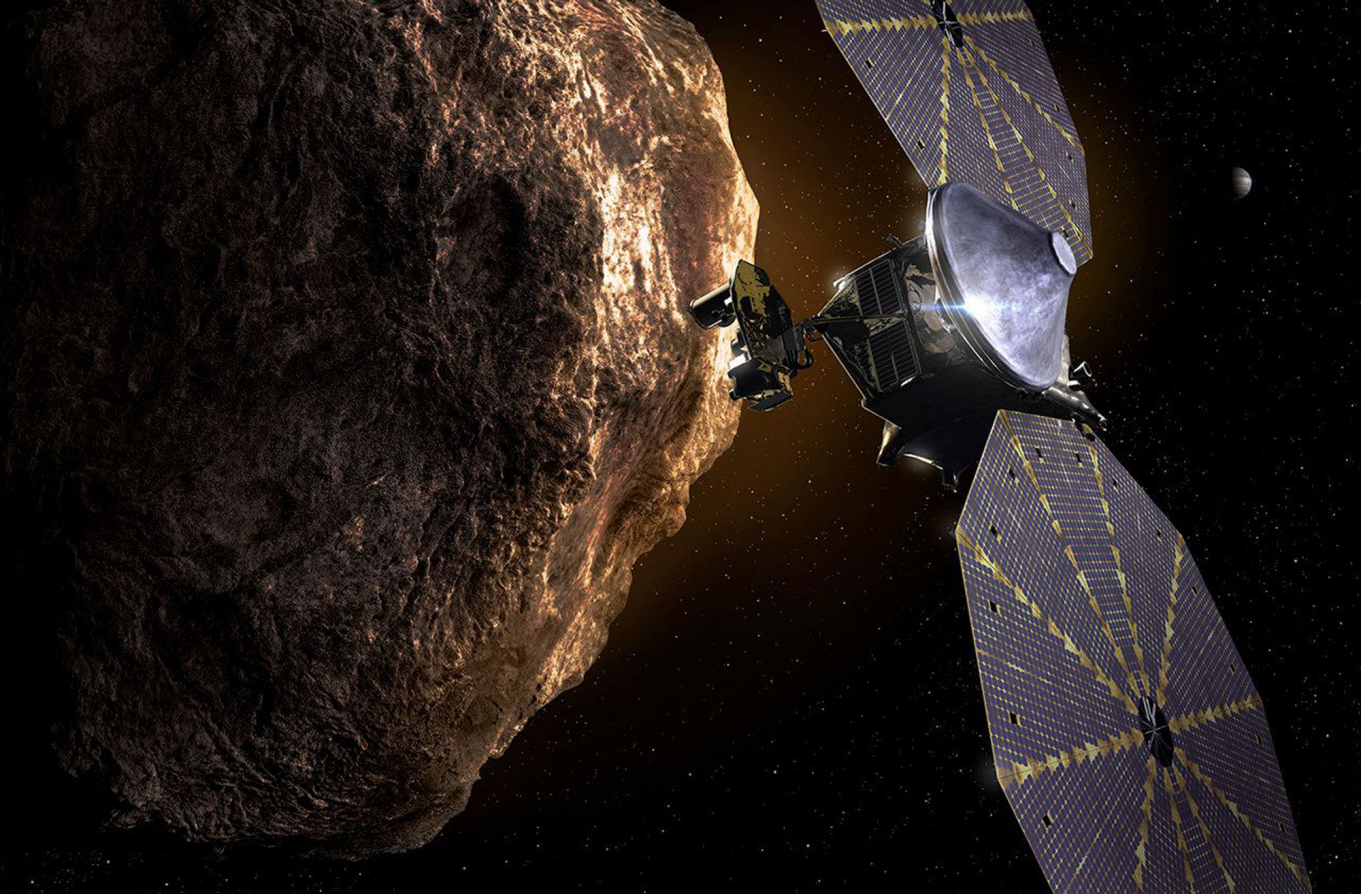
Southwest Research Institute.

In Christopher Nolan’s ‘Interstellar’, one character noted that our generation was born too late to explore Earth, and too early to explore space – yet it feels we might be just in the perfect sweet spot to explore our Solar System origins. It remains to be seen whether NASA’s *Lucy* can live up to its Latin meaning and actually become a *light-bringer*. The Jovian system itself may hold the keys to unlocking these questions of origins to some extent either way. Jupiter hosts Europa, an icy-ocean moon, which could theoretically offer the possibility of hosting primitive life forms, and will be visited by NASA’s Clipper mission in the early 2030s. Appropriately, just like *Lucy*, also the Roman king of gods, *Iuppiter*, corresponds to the meaning of light in its indo-european linguistic roots—the Jovian system might indeed shine a major light on our questions of origin. Exciting times lie ahead of us!
